# C13-pyruvate administration revealed differential metabolism between heart, liver and red blood cells and improved heart function during endotoxemia

**DOI:** 10.1186/cc10623

**Published:** 2012-03-20

**Authors:** RM Bateman

**Affiliations:** 1 Keio University, Tokyo, Japan

## Introduction

The systemic inflammatory response to bacterial infection, or sepsis, results in a hypermetabolic state; yet, systemic metabolic changes in metabolism and the metabolic interaction between tissues and red blood cells are not well understood. The objective of this study was to assess changes in intermediary metabolism during the onset of an animal model of sepsis by determining glycolytic, TCA and PPP metabolites, amino acids and ATP levels in heart, liver and red blood cells.

## Methods

C57BL/6 mice (30 to 35 g) were injected intraperitoneally with lipopolysaccharide (LPS, 40 mg/kg) to induce endotoxemia. Six hours post LPS, C13-pyruvate (a key intermediate metabolite) was administered subcutaneously for fluxome analysis of intermediate metabolites. At 20, 40 and 60 minutes, heart, liver and red blood cells were collected and stored at -80°C. Labeled metabolites were measured using capillary electrophoresis-mass spectrometry, quantified by calculating the AUC/t0-60 and expressed relative to control. Heart function was monitored by echocardiography.

## Results

Red blood cells preferentially metabolized pyruvate (ninefold increase) compared to heart (1.2-fold increase) or liver (-2.1-fold decrease), and were a net lactate source (2.1-fold increase). Glycolytic intermediates increased in the heart, but decreased in red blood cells, while TCA intermediates decreased in the heart and amino acids increased in the liver. Under the hypoglycemic conditions of the animal model, red blood cells were found to accumulate glycerol-3-phosphate (red cell glycerol flux remained normal) and 2,3BPG following C13-pyruvate injection. ATP was stable in the heart, but decreased in the liver and red blood cells. Echocardiography revealed a transient recovery of left ventricular function that correlated with shifts in red blood cell metabolism.

## Conclusion

Metabolic investigation of different septic tissues revealed shifts in metabolism between organs, suggesting that sepsis induces complex metabolic shifts in response to changing nutrient availability and cell function; moreover, enhancing red blood cell metabolism may be beneficial to depressed organ function during the onset of endotoxemia.

